# Exploring the Ecological Implications, Gastronomic Applications, and Nutritional and Therapeutic Potential of *Juglans regia* L. (Green Walnut): A Comprehensive Review

**DOI:** 10.3390/nu16081183

**Published:** 2024-04-16

**Authors:** Shaikh Ayaz Mukarram, Sangram S. Wandhekar, Abdelhakam Esmaeil Mohamed Ahmed, Vinay Kumar Pandey, Oláh Csaba, Daróczi Lajos, Prokisch József, Endre Harsányi, Kovács Bela

**Affiliations:** 1 Faculty of Agriculture, Food Science & Environmental Management, Institute of Food Science, University of Debrecen, Böszörményi út 138, 4032 Debrecen, Hungary; ahmed.abdelhakam@agr.unideb.hu (A.E.M.A.); kovacsb@agr.unideb.hu (K.B.); 2 Doctoral School of Nutrition and Food Sciences, University of Debrecen, Böszörményi út 138, 4032 Debrecen, Hungary; 3 Young Scientist, World Food Forum, I-00100 Rome, Italy; 4 Department of Food Engineering, College of Food Technology, Vasantrao Naik Marathwada Krishi Vidyapeeth, Parbhani 431402, Maharashtra, India; 5 Faculty of Forestry, University of Khartoum, Khartoum North 13314, Sudan; 6 RDC, Biotechnology Department, Manav Rachna International Institute of Research and Studies, Faridabad 121004, Haryana, India; v.k.pandey30@gmail.com; 7 Department of Neurosurgery, Borsod County Teaching Hospital, 3526 Miskolc, Hungary; olahcs@gmail.com; 8 Y-Food Ltd., Dózsa György út 28/A, 4100 Berettyóújfalu, Hungary; daroczi@yfood.hu; 9 Faculty of Agriculture, Food Science and Environmental Management, Institute of Animal Science, Biotechnology and Nature Conservation, University of Debrecen, Böszörményi út 138, 4032 Debrecen, Hungary; jprokisch@agr.unideb.hu; 10 Faculty of Agriculture, Food Science and Environmental Management, Agricultural Research Institutes and Academic Farming (AKIT), University of Debrecen, Böszörményi út 138, 4032 Debrecen, Hungary; harsanyie@agr.unideb.hu

**Keywords:** green walnut, therapeutic uses, culinary applications, ecological aspects, circular economy

## Abstract

The green walnut, which is frequently overlooked in favor of its more mature sibling, is becoming a topic of great significance because of its unique ecological role, culinary flexibility, and therapeutic richness. The investigation of the bioactive substances found in green walnuts and their possible effects on human health has therapeutic potential. *Juglans regia* L. is an important ecological component that affects soil health, biodiversity, and the overall ecological dynamic in habitats. Comprehending and recording these consequences are essential for environmental management and sustainable land-use strategies. Regarding cuisine, while black walnuts are frequently the main attraction, green walnuts have distinct tastes and textures that are used in a variety of dishes. Culinary innovation and the preservation of cultural food heritage depend on the understanding and exploration of these gastronomic characteristics. Omega-3 fatty acids, antioxidants, vitamins, and minerals are abundant in green walnuts, which have a comprehensive nutritional profile. Walnuts possess a wide range of pharmacological properties, including antioxidant, antibacterial, antiviral, anticancer, anti-inflammatory, and cognitive-function-enhancing properties. Consuming green walnuts as part of one’s diet helps with antioxidant defense, cardiovascular health, and general well-being. *Juglans regia* L., with its distinctive flavor and texture combination, is not only a delicious food but also supports sustainable nutrition practices. This review explores the nutritional and pharmacological properties of green walnuts, which can be further used for studies in various food and pharmaceutical applications.

## 1. Introduction

The most common tree nut in the world is the walnut (*Juglans regia* L.). The tree is frequently referred to as an English, Persian, white, or common [1]. *Juglans regia* is its scientific name, and it is a member of the *juglandaceae family*. This species of walnut tree is indigenous to the Old World. It originated in an area that stretches from the Balkans eastward to the western Himalayan chain and was grown in Europe as early as 1000 BC [2]. Currently, walnuts are farmed for commercial purposes in the USA, western South America, eastern Asia, northern Africa, and southern Europe. The top producer in the world is China, followed by the United States, Iran, Turkey, Romania, Ukraine, France, and India [3]. Walnuts harvested before they reach full maturity are known as green walnuts. When they were still green and the shell had not yet hardened, they were harvested from the tree. The exterior shells of green walnuts are soft and green. The nut is not fully hardened; it still forming inside [4]. Compared to fully grown walnuts, green walnuts have a unique flavor profile that is milder and less intense than that of fully mature walnuts. They are frequently described as having a slight citrus or tangy flavor. Depending on the region and temperature, green walnuts are normally harvested in late spring or early summer, usually in June or July. They are listed as priority plants by the FAO because of their high nutritional value. They are grown commercially worldwide. They are referred to as high-density nutritional nuts because of their high protein and fatty acid contents. Recently, there has been an increase in the production of walnuts that are valued for their nutritional content. Walnuts contain high concentrations of calories, proteins, carbohydrates, fat, minerals, and vitamins. They are well known as one of the most vital nuts globally. Walnuts are very healthy and a mainstay of the Mediterranean diet because of their nutrient-dense nature. Walnuts have drawn considerable attention because of their health-promoting properties [5]. They have a wide range of pharmacological properties, such as antioxidant, antibacterial, antiviral, anticancer, and anti-inflammatory activities, and can aid in cognitive function improvement and reduce immunotoxic effects [6]. In traditional medicine, they have been used to treat prostate and cardiovascular diseases, inflammation, diarrhea, cancer, and inflammation. Numerous studies have indicated that whole walnut plants are beneficial for treating a variety of illnesses. The nut extract prevented photoaging, inflammation, cancer growth, oxidative damage, and wrinkles. Dried walnut shells have been used as media to separate water and crude oil as well as for polishing jewelry, metal, and gun casings [7].

Walnuts are a good source of flavonoids, polyphenols, flavanols, carbohydrates, fatty acids, cardiac glycosides, steroids, minerals, tannins, proteins, dietary fiber, melatonin, plant sterols, α-tocopherol, folate, tannins, vitamin A, vitamin C, and vitamin E family compounds [8]. The antibacterial activity of phenolic extracts has been demonstrated in numerous studies, making them ideal alternatives to antibiotics and food preservatives. Professionals in the food industry believe that this natural substance has significant economic value. The product is prized for its nutritional value, health benefits, and mouthfeel, and it is popularly enjoyed as royal cuisine worldwide. Because of its hard, fibrous structure, the dried bark of walnut trees has been used as a brush for mechanical tooth cleaning. Juglone is the primary and most significant component. It is regarded as an efficient tooth-whitening, anti-plaque, antifungal, antibacterial, and anticariogenic substance in dentistry. In an effort to provide better dental care, scientists and researchers have been interested in researching the chemicals and compounds found in bark in recent years [9]. In a comparison of walnut fruit green husk extract with contemporary semi-synthetic and commercial hair dyes, it was found to be an effective natural hair-coloring agent with the highest level of antimicrobial action. It can be utilized as a safe, valuable, affordable, and environmentally acceptable source of dyes [10]. Various value-added and healthy food products have been formulated from green walnuts, including pickles, jams, baked products, and beverages. Green walnut extracts are used for fortification and enrichment. The present review reports updated knowledge on their nutritional constituents, health and medicinal benefits, ecological impact, culinary uses, environmental impact, sustainability, various applications, and prospects that have great significance for the welfare of humankind.

## 2. Current Research on Green Walnut and Its Availability

**Figure 1:**
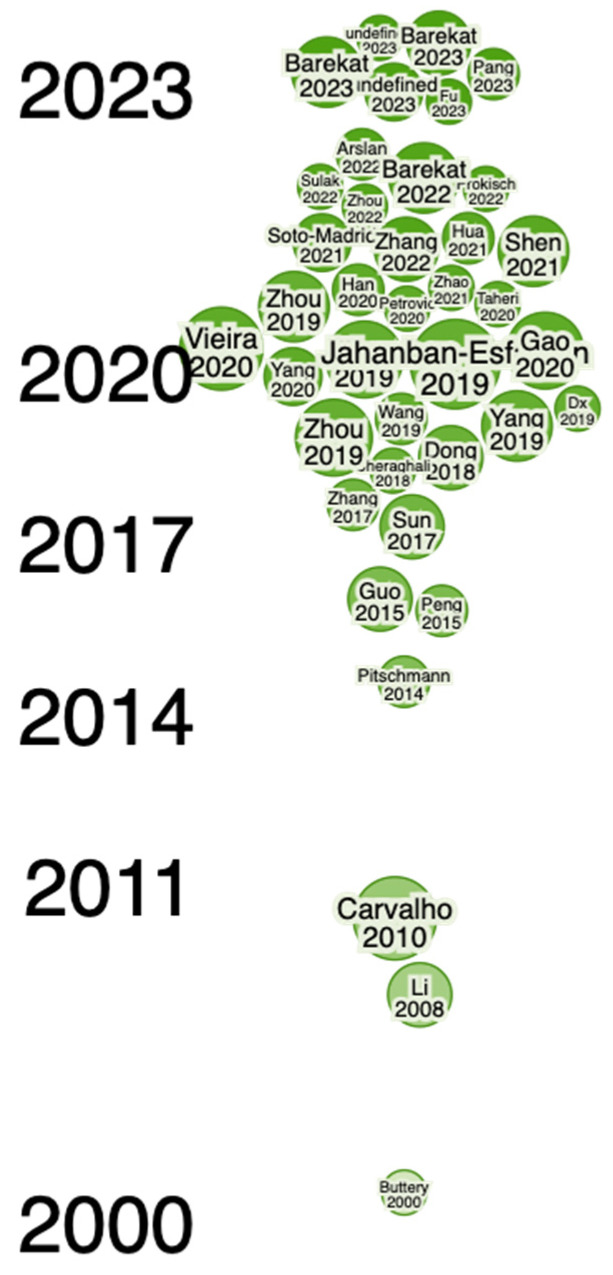
The current availability of articles (timeline of Scopus- and WoS-indexed articles) from 2011 to 2023 (40 articles) which give insights into research conducted on *Juglans regia* L. (green walnut) and its different therapeutic and other uses (in green color).

**Figure 2:**
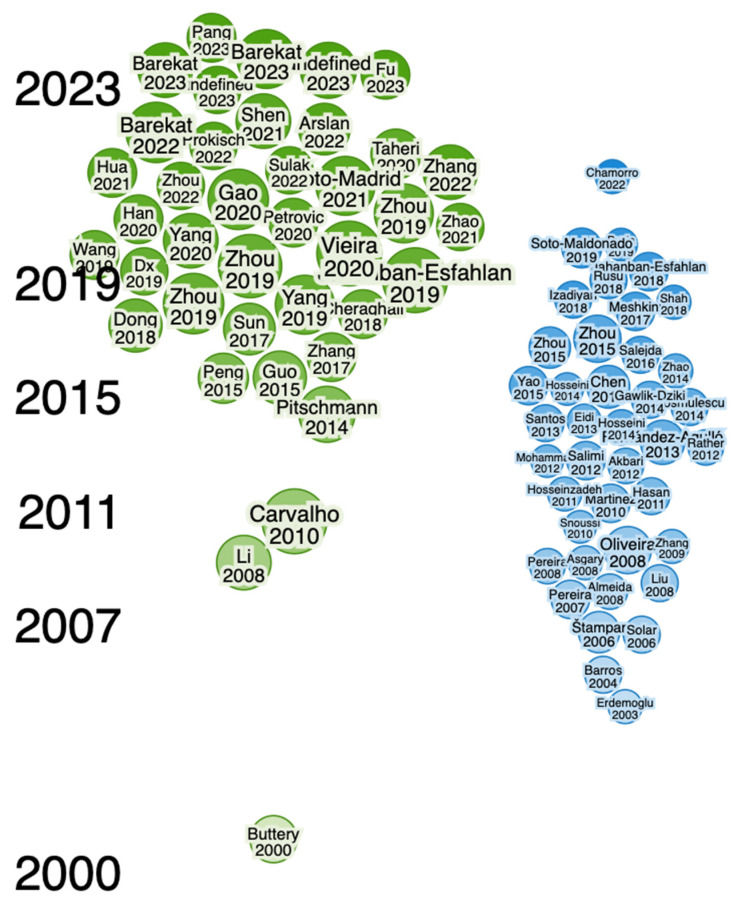
Similarities between work conducted on *Juglans regia* L. (green walnut) and different available articles related to its own properties and characteristics, etc. (in blue color) (from 2007 to 2023).

**Figure 3:**
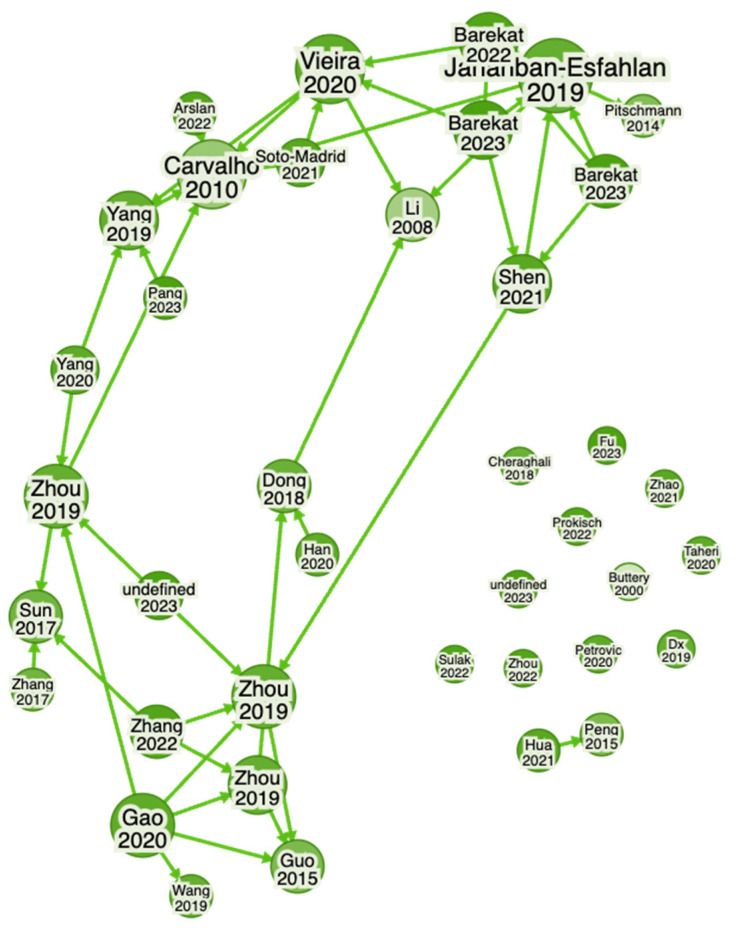
Network of the current availability of articles by different authors (network of Scopus- and WoS-indexed articles) from 2011 to 2023 (40 articles), which gives insights into research conducted on *Juglans regia* L. (green walnut) and its different uses. (in green color).

**Figure 4:**
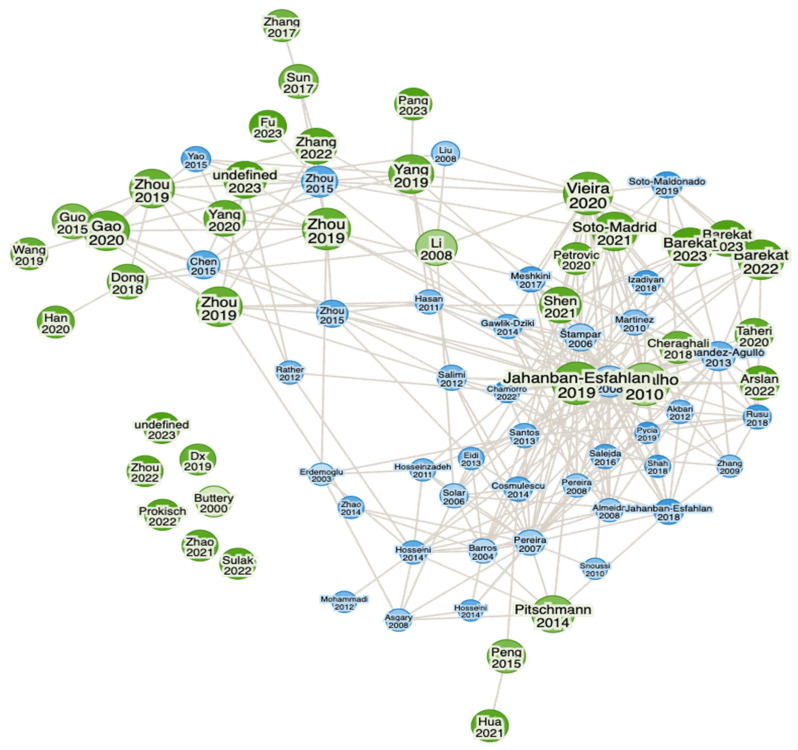
Network between authors and their works conducted on *Juglans regia* L. (green walnut), with different available articles which are related to its own properties and characteristics, etc. (in blue color).

Newly developed software and online tools were used to identify key research findings about green walnuts through the Web of Science (WoS) and Consensus (an AI-based search engine) databases to find literature and key findings available online via different databases. To find gaps and explore the identified authors, collaborations, summaries, etc., in detail, Research Rabbit (an AI-powered search engine) was used. The following terms were used to search for findings on green walnuts: “Green Walnut, *Juglans regia* L. (Green Walnut), Benefits of Green walnut in Medicinal uses, Therapeutics uses, Culinary uses, Ecological uses”. It uncovered close connections of articles published during the period described below and shows that the articles were published from 2011 to 2023 and from 2007 to 2023 (Figure 1, Figure 2, Figure 3 and Figure 4). Approximately 40 of them were related to its use in therapeutics and other applications, whereas approx. 40 articles were related to its morphology and characterization.

## 3. Botanical Profile and Impact

**Figure 5:**
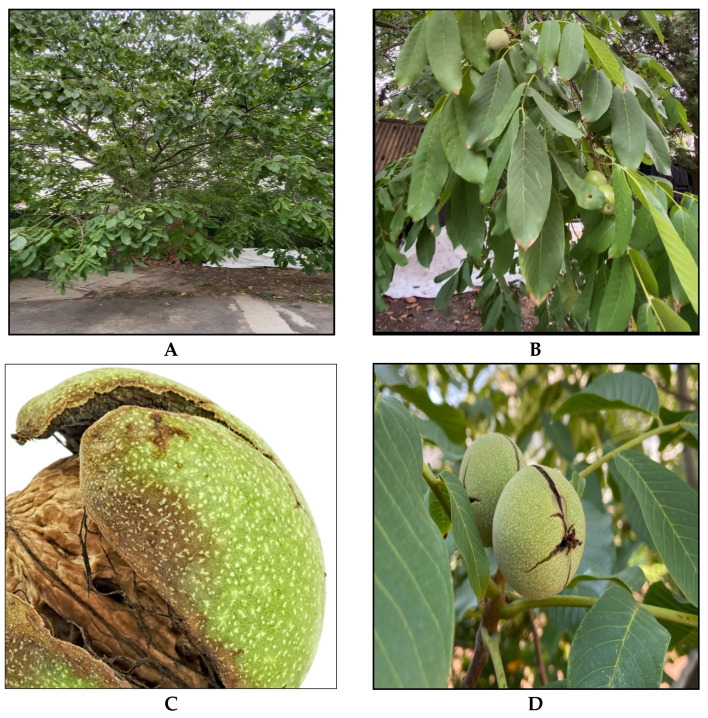
Real-time images of (**A**) green walnut tree, (**B**) leaves, (**C**) husk, and (**D**) green walnut fruit.

**Figure 6:**
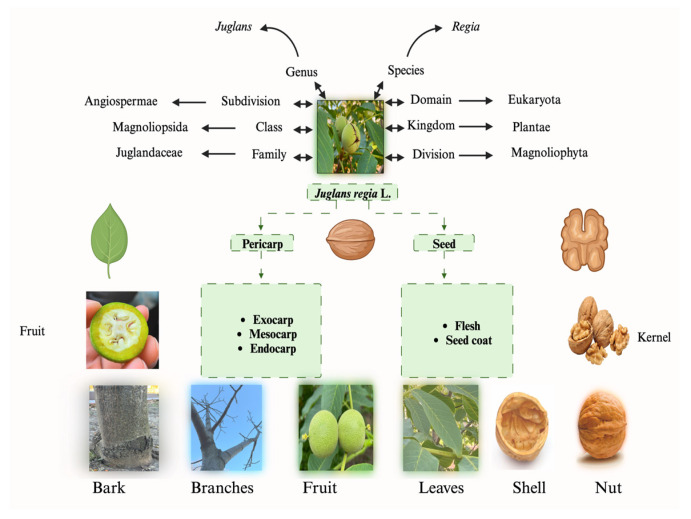
Taxonomic classification of *Juglans regia* [12,13] (created using BioRender.com).

Walnut (*Juglans* spp.) belongs to the family *Juglandaceae*, which consists of seven genera comprising approximately 60 monoecious tree species [11]; it is of the order Fagales and is a diploid species with a somatic chromosome number (2n = 32). The genus *Juglans* comprises 21 species [12]. Here, Figure 5 shows real-time images of walnut trees and their parts grown on the campus of the (Faculty of Agriculture, Food Science and Environmental Management, University of Debrecen). The taxonomic classification of *Juglans regia* is shown schematically in Figure 6.

Walnuts are monoecious in nature, with male and female flowers borne at different locations on the tree [14]. Male flowers develop laterally from simple buds on 1-year-old wood, while female flowers generally develop in the panicle of 1–3 flowers from mixed buds in which flowering takes place at the terminal position of the current season’s leafy shoot. Based on the position of the emergence of leafy shoots, walnut cultivars can be classified into three bearing groups: terminal, intermediate, and lateral [15]. The fruit-bearing habit is associated with branching density, the position of flowering buds on annual shoots, and age of the fruiting shoot bearer [16]. Each group of walnut variety differs from the others in terms of the number of branches on its 1-year-old shoot. The bearing habit also influences the tree’s structure and productivity [17]. Lateral bearing is associated with precocity and a higher yield. Lateral bud fruitfulness is considered the most significant yield factor and can also be manipulated through breeding [18]. There is a need to select varieties with desirable traits, such as lateral bearing, dense branching, short stature, earliness in bearing, good nut size, and a better nut–kernel ratio. Large germplasm collections, the extent of variation in these collections, and their accessibility to biologists and breeders are essential factors affecting their utilization in crop improvement programs.

## 4. Best Practices for Harvesting of Green Walnuts

Harvesting green walnuts involves collecting young, meristematic walnuts directly from trees before their shells form. This sustainable practice can yield delicious results in various recipes [19].

Here are some best practices for harvesting green walnuts:

### 4.1. Harvesting Time

Green walnuts are typically harvested from late May to the end of June, when they are easy to cut open and not yet mature. Early June is a good time to check walnuts by picking them and cutting them open [20].

### 4.2. Selecting the Right Nuts

The selected green walnuts were approximately the size of a small ping-pong ball, easy to cut, and not fully grown.

### 4.3. Wearing Gloves

Green walnuts can stain everything; therefore, it is essential to wear gloves while harvesting and processing them.

### 4.4. Picking Method

Walnuts are harvested from the tree in a manner similar to other fruits and then placed in a bag. Some people use a ball-style walnut harvester or a rolling nut picker to make the process more efficient.

### 4.5. Processing

Green walnuts can be used in various recipes, processed products, and as nutraceuticals. “It is recommended to use walnuts harvested early to ensure that the inner shell is edible and easy to cut”. It is important to wear dark clothing and gloves during processing to avoid staining [21]. In addition, there is a need for some pre-treatments, such as soaking in water, to remove bitterness.

### 4.6. Storage

Green walnuts can be stored in cold, dark spaces for up to a week before processing. Green walnuts can be processed and preserved in the form of slices or powder. The conservation of green walnuts involves a combination of environmental initiatives, preservation methods, the protection of walnut tree species, community projects, and sustainability efforts in walnut farming. These approaches contribute to the preservation of natural resources and the promotion of greener environments [20]. The utilization of various parts of green walnut is shown in Table 1, showing their uses and the availability of nutrients and bioactive compounds; Table 2 shows the nutritional information of green walnut.

### 4.7. Effect of Geographical Origin of Walnuts on Their Functionality and Composition

The geographic origin of walnuts (*Juglans regia* L.) can greatly impact their chemical composition and usefulness. The variety of walnut genetic variants in various places leads to differences in forestry, productivity, and nut attributes such as physical and chemical features [22]. A study of six walnut cultivars growing in Portugal found variations in their chemical makeup, including moisture, total oil content, crude protein, ash, carbon, and nutritional value [23]. The cultivars showed differences in their fatty acid and sterol profiles, with polyunsaturated fatty acids, particularly linoleic acid, being the most abundant [23]. Walnuts can have an oil concentration as high as 740 g/kg in certain commercial types, making it a significant nutritional component of the nut. Walnut oil is primarily composed of unsaturated fatty acids such as linoleic, oleic, and linolenic acids. Additional elements, such as tocopherols, phospholipids, sphingolipids, sterols, hydrocarbons, and volatile substances also influence the characteristics of the oil. The amount of phenolic chemicals in the seed coats of walnuts, which possess potent antioxidant qualities, fluctuates and is determined by the genetic makeup and geographical location of the walnuts [3]. Portuguese walnut cultivars exhibit variations in oxidative stability, peroxide values, and sterol profiles which are crucial for the shelf life and nutritional quality of walnut oil [23]. Ultimately, the geographic source of walnuts plays a crucial role in determining their chemical makeup and usefulness, impacting aspects such as nutritional value, oil content, fatty acid and sterol compositions, and antioxidant properties. These differences can be explained by the genetic diversity of the various walnut cultivars and the environmental conditions in which they are grown.

**Table 1:** Uses of different parts of green walnut (including bioactive compounds and medicinal benefits).

Part of the Plant	General Uses	Bioactive Compounds		Medicinal Benefits	Availability of Nutrients	References
Kernel	Used as a traditional remedy for the treatment of stomachache and cough.	Flavonoids	(+)-catechin Hydrate; (+)-catechin; (−)-epicatechin; Rutin;gallate (+)-catechin hydrate quercetin; syringol; chrysin; myricetin	Hypolipidemic effect; antioxidant activity; antibacterial properties; antiproliferative properties; chemo-preventive properties	A rich source of mono- and polyunsaturated fatty acids; also contains a high number of phenolic compounds.	[3,22,24,25,26,27]
		Tannins	Quercetin-3-ß-d- glucoside 1;3;6- trigalloylglucose penta-o-galloyl-ß-d- glucose			
		Phenolic acids	Neochlorogenic acid; ferulic acid;vanillic acid; syringic acid; caffeic acid; chlorogenic acid; ellagic acid; p-coumaric acid; cinnamic acid; quinic acid; gallic acid p-hydroxybenzoic acid			
		Quinones	-			
Husk	The green husk is used in the preparation of liqueur; the husk extract as used as a source of antioxidants to suppress the level of LDL cholesterol and to treat and relieve pain related to skin disorders.	Flavonoids	Apigenin; apigenin; 7-o-b-d-glucuronide; rutin; kaempferol; (+)-catechin; (−)-epicatechin; Myricetin; sudachitin; cirsilineol; 5;6;4′-trihydroxy-7;3-dimethoxy-flavone; eriodictyol	Liver- and kidney-protective; antioxidant, anti-inflammatory, and anticancer properties; improves immune function	Contains gallic acid, ellagic acid, and flavonoids.	[28,29,30,31]
		Tannins	tannic acid;ellagic acid			
		Phenolic acids	n-hexadecanoic acid; octadecanoic acid; acetic acid; propanoic acid; formic acid; hydroxy-butanoic acid; pentanoic acid; crotonic acid; oxo-pentanoicacid;tetradecanoic acid;2-methyl-propanoic acid; butanoic acid			
		Quinones	2-methoxy juglone (26.75) juglone (113.63) 8-dihydroxy-1;4- naphthoquinone; 2-hydroxy-14 Naphthoquinone; 8-hydroxyquinoline; 1;4- Naphthoquinone; juglone1-naphtho			
Shell	The shell is biodegradable in nature and helps improve soil health; it is used as mulching material to control weed growth and regulate the temperature of soil, and a powdered form of the shell is used as a filter medium.	Juglone; tannins; phenolic compounds; pyroligneous acids		Antioxidant, anticancer, and anti-inflammatory properties	Good source of micronutrients including vitamins and minerals; also exhibits oleophilic (oil-attracting) and hydrophobic (water-repelling) properties.	[19,32,33,34,35,36,37]
Bark	Walnut bark is used for cleaning teeth; bark paste is useful in the treatment of arthritis, skin diseases, and toothache and the growth of hairs.	Polyphenols; flavonoids		Prevents tooth ache and tooth decay; antimicrobial activity; antioxidant activity; antifungal activity; platelet aggregation; antiseptic	Walnut bark contains potassium hydroxide which has whitening properties, it is also rich in phenolic antioxidants.	[38,39,40,41,42]
Root	Root extracts of walnut havie inhibitory activities against pathogenic bacteria (*Escherichia coli*, *S. aureus*, *Staphylococcus saprophyticus*, *Klebsiella pneumonia*, and *Pseudomonas aeruginosa*).	Alkaloids; flavonoids; polyphenolic compounds		Hair loss; dandruff; skin disorders	Ethyl acetate extract, methanolic extract, and hexane extract have bacterial inhibition activity (phenolic compounds; saponins).	[41,43,44,45]
Leaves	Walnut leaves are utilized in traditional medicine as remedies to treat venous insufficiency and hemorrhoidal symptomatology, and for their anthelmintic, depurative, and antidiarrheal effects. Dried walnut leaves are used as an infusion.	Phenolic acids; tannins; essential fatty acids; ascorbic acid; flavonoids; caffeic acid; para-coumaric acid; Juglone; galactosidase; arabinoside; xyloside; rhamnoside; Naphthoquinone		Antioxidant activity; lipid-lowering effect; antihypertensive effect; antimicrobial effects; gastroprotective activity; hypercholesteraemic activity; antidiabetic effect; anticancer effect; hepato-protective activity and aanti-aging activity	Juglone is a natural phenolic compound is found in fresh walnut leaves. Walnut leaves are also good source of flavonoids, ascorbic acid, and p-coumaric acid.	[46,47,48,49,50,51]

**Table 2:** Nutritional information of green walnut.

Nutritional Component	Percentage	Pharmaceutical Potential	Impact on Human Health	Reference
Vitamin C	8.7 mg/100 g	Antioxidant; immune system support	Helps protect cells from damage; boosts immune system	[52]
Vitamin E	2.6 mg/100 g	Antioxidant; anti-inflammatory	Helps protect against oxidative stress; reduces inflammation	[53]
Omega-3 Fatty Acids	--	Anti-inflammatory; cardiovascular support	Supports heart health; reduces inflammation	[54]
Polyphenols	--	Antioxidant; anti-inflammatory	May help reduce inflammation; protect against chronic diseases	[55]
Melatonin	--	Sleep regulation; antioxidant	Helps regulate sleep; acts as an antioxidant	[53]
Fiber	3.4 g/100 g	Digestive health; weight management	Aids in digestion; helps maintain a healthy weight	[56]
Minerals (e.g., Magnesium, Potassium)	--	Muscle function; bone health	Important for muscle and nerve function and bone health	[57]

## 5. Different Applications of Walnut in Medicine and Gastronomy

### 5.1. Antitumor Activity

**Figure 7:**
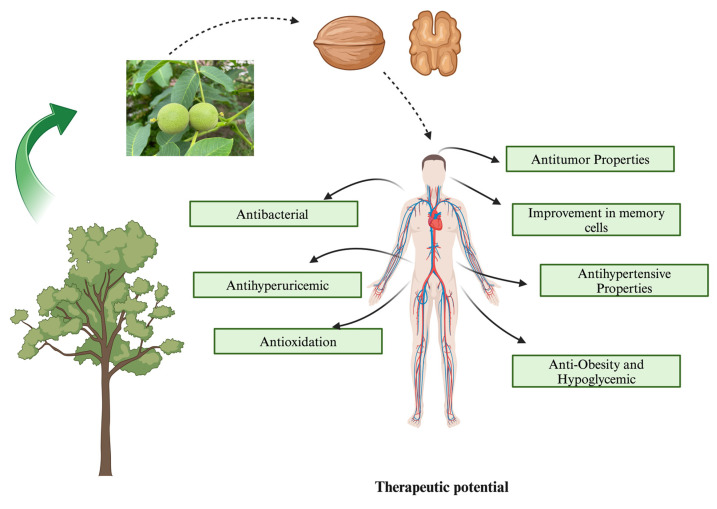
Therapeutic potential of *Juglans regia* L. (green walnut) [52,53,54,55,56,57] (created using BioRender.com).

Green walnut husk extract and nanotechnology have been shown to have antitumor effects in several studies. Among them, green synthetic silver nanoparticles (Ag-NPs) significantly improved antioxidant activity and cytotoxicity against cancer cell lines [58]. Researchers have discovered that the temperature used during preparation is a critical component in the production of Ag-NPs using a WGH extract. Furthermore, at concentrations below 250 μg/mL, Au-NPs had minimal toxic effects on both normal and cancer cells, making them appropriate for a range of medicinal uses owing to their dose-dependent toxicity [59]. The impact of a WSP extract on leukopenia induced by chemotherapy and/or radiation therapy for cancer has also been studied. These findings demonstrated that the WSP extract promoted leukopenia and enhanced bone marrow development, differentiation, and division in mice [60]. They also examined how the WSP extract affects glioblastoma. They discovered that the WSP extract had a pro-apoptotic effect on glioblastoma and decreased tumor migration and multiplication. This outcome offers a new approach to treating tumors [53]. Walnut materials contain a wide variety of bioactive peptides, including xanthine oxidase inhibitory, antioxidant, and antihypertensive active peptides [61,62]. As Shown in Figure 7.

### 5.2. Improving Memory Loss/Improving Cells/Antihypertension

**Figure 8:**
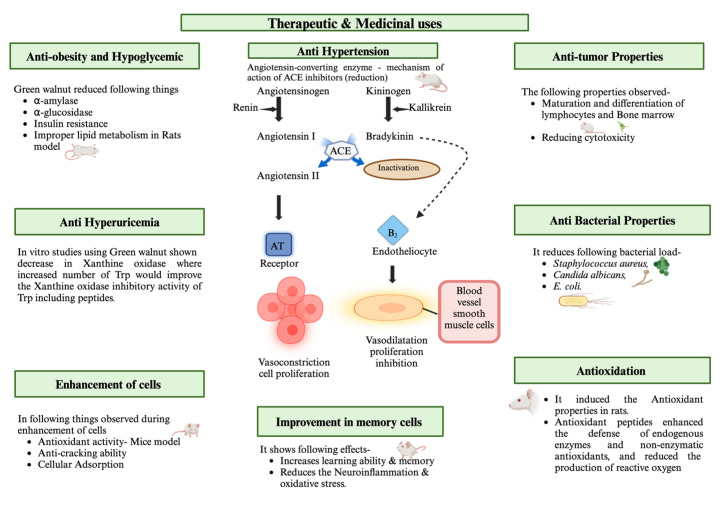
Therapeutic and medicinal properties of *Juglans regia* L. (green walnut) (created using BioRender.com).

Green walnuts have been shown to reduce neuroinflammation, balance the cholinergic system, reduce oxidative stress, and promote autophagy, all of which have a positive effect on the symptoms of Alzheimer’s disease, Parkinson’s disease, and memory impairment [63]. Green walnuts were discovered to reduce oxidative stress and modify the cholinergic system; WMBPs might ameliorate scopolamine-induced memory impairment in mice. They also discovered that two WM neuroprotective peptides, FY and SGFDAE, were effective in ameliorating memory impairments [64]. Furthermore, three peptides (GGW, VYY, and LLPF) from WM were found to have powerful neuroprotective effects in glutamate-treated PC12 cells. These peptides prevented Ca^2+^ influx, collapsed the mitochondrial membrane potential, and controlled the expression of proteins linked to apoptosis Furthermore, three peptides (GGW, VYY, and LLPF) from WM were found to have powerful neuroprotective effects on glutamate-treated PC12 cells. These peptides prevent Ca^2+^ influx, collapse the mitochondrial membrane potential, and control the expression of proteins linked to apoptosis [65]. Later, it was shown that WMBPs may have a neuroprotective effect on neurotoxicity caused by aluminum chloride and d-galactose in mice. The findings showed that WMBP treatment improved learning and memory in these mice by reducing oxidative stress and reversing cholinergic dysfunction [66]. As shown in Figure 7 and Figure 8.

They are also used to produce various healthy and nutritional products. When consumed as part of a nutritious diet, walnuts, including green walnuts, can provide a variety of benefits such as improving heart health and supporting optimal brain function. Green walnuts are also an excellent source of omega-3 fatty acids, specifically alpha-linolenic acid, which boost memory, concentration, and cognitive function. Green walnuts are used for culinary and medical purposes, and their health benefits are immense. It is worth mentioning that green walnuts are a high-calorie food, but they are also satiating and offer a diverse range of nutrients. Therefore, they can be guilt-free snacks for those not on a strict calorie-deficit diet. The hulls of green and black walnuts are excellent sources of essential nutrients and have strong parasite-cleansing benefits. Walnuts have a long history of use in folk medicine. Although the use of synthetic and chemical drugs has increased over the past 50 years, the negative side effects of these drugs have caused an increase in naturally based drugs. Drugs derived from plants have long been among the most successful approaches. Studies have shown that walnuts increase blood levels of high cholesterol (HDL) and protect against cardiovascular diseases [67]. Moreover, walnuts have been suggested to prevent the onset of severe illnesses, such as Parkinson’s disease and Alzheimer’s disease. Eating walnuts can increase blood melatonin concentrations [68]. The development of intelligence in children is positively affected by walnuts. They benefit the human digestive system because of their high fiber content. They can aid in recovery from conditions such as sleep disorders [69]. The medicinal properties of green walnuts are shown in Figure 8.

Hypertension is a prevalent cardiovascular condition that is a significant risk to human health [70]. ACE is a crucial enzyme in the blood-pressure-regulating system, and its activity can be altered by ACE-inhibitory peptides. All ACE inhibitors can efficiently prevent the conversion of angiotensin I to angiotensin II, resulting in comparable therapeutic and physiological outcomes. WM is a common source of ACE-inhibitory peptides. A previous study found that a WM protein hydrolysate exhibited potent ACE-inhibitory action and stability, leading to a substantial decrease in systolic blood pressure in hypertensive mice [71]. Furthermore, another study utilized alcalase and trypsin for the hydrolysis of WM. The hydrolysate showed significant ACE-inhibitory action and low IC50 values [72], as shown in Figure 8 above.

### 5.3. Regulation of Blood Sugar/Anti-Obesity and Hypoglycemic Effect/Antihyperuricemia

Studies conducted in modern pharmacies have shown that walnuts improve protein synthesis, control liver function, exhibit anti-allergic qualities, control blood sugar, and regulate blood circulation [73]. Walnuts have been used for years as kidney stone removers, weight gain aids, as anti-vomiting agents during pregnancy, and for their calming [74]. Walnut leaves and shells, in addition to the inside of the fruit, have long been utilized in complementary and alternative medicine [75]. The pharmacological properties of walnut leaves include hypoglycemic effects, antifungal activity, diarrhea suppression, and skin-cleaning properties. Moreover, vascular protection and tumor inhibition have been reported to be beneficial. Owing to their antiseptic qualities, walnut leaves are used in both alternative and modern medicine to treat skin inflammation and ulcers [76]. Walnut leaves have also been applied externally to treat bites from bees, wounds, inflammatory skin conditions, hand sweating, and foot sweating.

### 5.4. Antibacterial Agents

Hydrothermal charcoal was created using walnut shells to combat Pseudomonas species, *Candida albicans*, *Staphylococcus aureus*, and *Klebsiella pneumoniae.* They discovered that walnut shell hydrothermal charcoal may destroy the cell structures of these bacteria, with *C. parapsilosis* showing maximum inhibition (96.67%). Therefore, walnut shell hydrothermal charcoal is a natural disinfectant that is less expensive, safer, and more environmentally friendly than chemical disinfectants [77]. Antibacterial agents are also abundant in green walnuts. A study reported that the highest levels of antioxidant and antimicrobial activities were observed in mature green walnut husks with open shells. Green walnut husks in different states, such as open and closed, exhibit varying antioxidant and antibacterial properties. However, independent of the condition of the green walnut husk, extracts were able to stop the growth of Gram (−) bacteria, such *Escherichia coli* [78]. Furthermore, a small number of studies have compared the effects of clotrimazole and green walnut extracts on *Candida albicans* in female rats. The findings demonstrated that after one week of treatment with the produced vaginal cream, the growth of *Candida albicans* was significantly suppressed in female rats. Additionally, the use of nanotechnology in our daily lives is becoming more widespread [79].

Silver chloride nanoparticles were synthesized using room-temperature WGH extracts. Subsequently, the synthesized particles were subjected to bacterial inhibition tests which demonstrated a notable inhibition of clinical isolates of *S. aureus* and *E. coli*. This investigation led us to hypothesize that food storage and bacterial inhibition would benefit from a combination of bioactive compounds and AgCl nanoparticles [80].

### 5.5. Enzyme Inhibitors

The by-products of walnuts have been shown to have potent enzyme-inhibiting effects in multiple investigations. This study investigated the effects on rats of a walnut seed extract cooked with or without a shell. Researchers discovered that the extracts of walnuts roasted in their shells shown better results in preventing the synthesis of ACE, phosphodiesterase-5, arginase, and acetylcholinesterase [81]. A small number of researchers observed that adding a WS extract to LPS-stimulated macrophages reduced the expression of AP-1, TNF-α, IL-8, iNOS, and COX-2 [82]. One of the main enzymes in the blood-pressure-regulating system is ACE, and ACE-inhibitory peptides can control ACE activity. The conversion of angiotensin I to angiotensin II can be successfully blocked by all ACE inhibitors, and their physiological and therapeutic effects are comparable. One popular source of ACE-inhibitory peptides is walnut extract. Still, research is ongoing to determine the effects of green walnut extract as an enzyme inhibitor.

### 5.6. Antioxidant Activity

Green walnuts contain potent antioxidants such as quercetin, juglone, and ellagic acid, which are associated with a lower risk of chronic diseases, including heart disease and certain cancers [83]. Walnuts are gaining popularity and importance in human nutrition because of their high antioxidant content, nutritional value, and ability to prevent oxidative stress and the damaging effects of free radicals [84]. Researchers have placed high value on identifying the bioactive compound content of green walnuts, determining their antioxidant activity, and evaluating the significance of these compounds for human health [28]. Green walnuts have a unique nutritional profile which makes them superior to their dry counterparts. Significant ratios of vital elements were found in 100 g of fresh walnuts according to a proximate composition study. Ash (3% ± 0.3), protein (16% ± 0.7), moisture (20% ± 0.5), fat (40% ± 0.2), and total carbohydrates (21% ± 0.2) were found, which makes it important [85]. Numerous bioactive substances, such as polyphenols, juglone, and vitamins (B6, folate, riboflavin, niacin, thiamine, and pantothenic acid), have been found in green walnuts [23]. Green walnuts are an excellent source of omega-3 fatty acids, specifically alpha-linolenic acid, which are essential for boosting memory, concentration, and cognitive function. A significant number of total polyphenols balance the high level of unsaturated fatty acids, which makes them susceptible to lipid peroxidation and increases their antioxidant capacity [28]. Juglone is a bioactive compound found in the green husk of walnuts and has the chemical formula 5-hydroxy-1,4-naphthoquinone [52]. It is sparingly soluble in hot water but is soluble in alcohol, acetone, chloroform, benzene, and acetic acid. The extraction of juglone from walnut green husks has been investigated using supercritical carbon dioxide and ethanol as a co-solvent [86].

### 5.7. Culinary Uses

**Figure 9:**
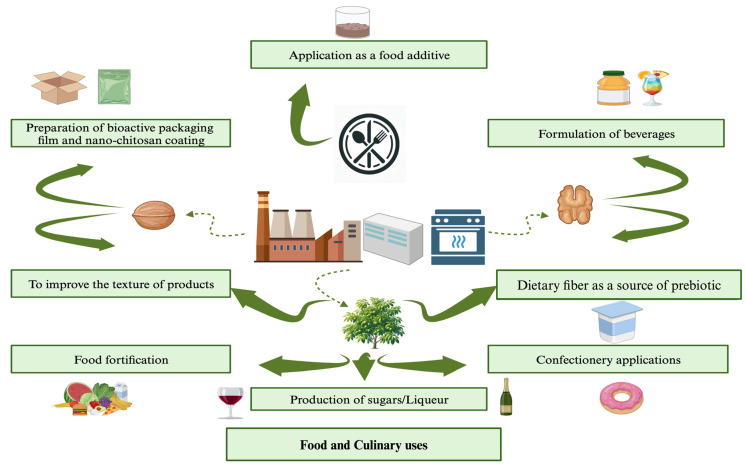
Food and culinary uses of *Juglans regia* L. (green walnut) (created using BioRender.com).

Green walnuts have a brightly spiced citrus scent and warm, spicy flavor, making them a popular ingredient in various culinary traditions. They are commonly used to produce condiments, pickles, sweets, preserves, and a variety of liqueurs [3]. Some of the culinary uses of green walnuts are shown in Figure 9.

#### 5.7.1. Nocino and Vin de Noix

Green walnuts can be used to make Nocino, a traditional Italian alcoholic beverage made by steeping green walnuts in alcohol with various spices and sugars, and vin de noix, a French walnut wine [87]. As mentioned in Table 3.

#### 5.7.2. Pickles and Preserves

Green walnuts can be used to make pickles and chutneys, and they can be preserved. When cured in brine and sun-dried, they become akin to fleshy, tart olives. Green walnut preservation is a popular practice in many countries, including Turkey, Iran, and Azerbaijan. It involves boiling green walnuts in sugar syrup until they become tender and sweet. The preserved walnuts can be enjoyed on their own or used as a topping for desserts [88].

#### 5.7.3. Food Additives

When processing and storing edible oils, green walnut husks are primarily utilized as natural antioxidants as substitutes for synthetic antioxidants [89]. This may enhance the organoleptic acceptability of the oil’s texture and odor. Additionally, it prolongs shelf life and prevents the growth of microbes during storage [90]. It also helps reduce the oxidation reaction in sunflower oil [89]. It can also be used as a natural antioxidant in various food applications [91]. As Mentioned in Table 3.

#### 5.7.4. Food Films/Alcohol

It has several benefits for making cling films and packaging materials that are safe for food. These can be called bioactive films that help protect fruit freshness and quality [92] and have also been used to make alcohol (liqueur) which has a high number of polyphenols that help stomachaches [56,87,93]. In addition, it can be used in several applications in food, such as baking and preparing sauces, pesto, and salads. There are a few examples of culinary innovations that use green walnuts. They are versatile ingredients that can add a unique flavor and texture to many dishes, from sweet desserts to savory foods, bread, beverages, and dips. There is an economic demand for green walnut in the food processing, nutraceutical, and medical fields because it contains several bioactive components and has a good nutritional profile that can be utilized for value addition and commercialization. As Mentioned, its overall uses in Table 3.

**Table 3:** Different uses of green walnut in medicinal, culinary and ecological applications [19,56,58,59,63,64,78,79,87,90,91,92,93,94,95,96,97,98,99,100,101,102,103,104,105,106,107,108,109,110,111,112,113,114,115,116,117].

Therapeutic Uses	Food/Culinary Uses	Ecological/Industrial Uses	References
Antibacterial agent	Application as a food additive	Contributes to increasing soil carbon	[78,79,90,91,118]
Antitumor activities	Preparation of bioactive packaging film and nano-chitosan coating	Helps in reducing pollution	[58,59,92,95,96,97]
Improves memory loss and protection of nerves	Formulation of beverages	Improves soil health	[19,56,63,87,93,98,99]
Anti-Obesity and hypoglycemia	To improve product texture	Acts as a natural insecticide	[99,100,101,102]
Neuroprotection	Dietary fiber as a source of prebiotic	Acts as a natural herbicide	[64,103,104]
Anti-inflammatory properties	Food fortification	Preparation of polymer composites	[103,105,106,107,108,109]
Improves cells	Confectionery applications	Natural dye source	[10,87,95,110]
Prevention of chronic diseases	Production of sugars	Removal of hazardous materials	[111,112,113,114,115,116]

## 6. Ecological/Industrial Applications

**Figure 10:**
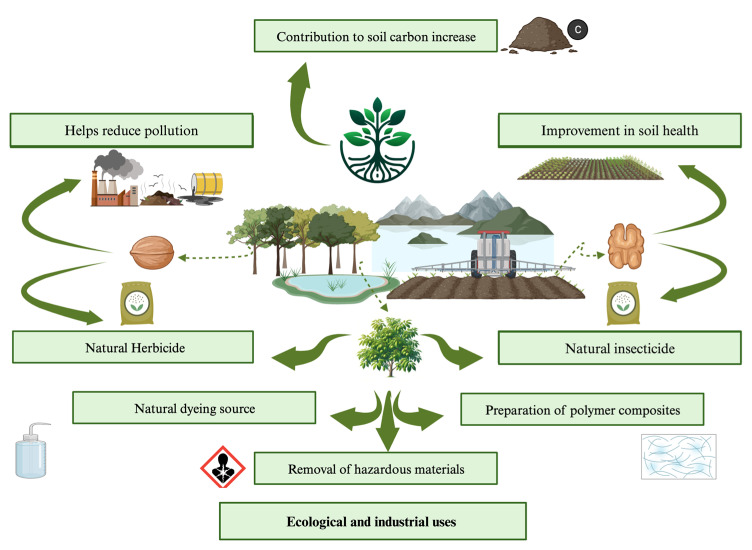
Ecological and industrial uses of *Juglans regia* L. (green walnut) (created using BioRender.com).

Walnut production is relatively sustainable, especially when organic. Compared with other nuts, walnuts have a low carbon footprint and use less land and water, making them one of the most sustainable nuts (Figure 10). Organic walnut production does not pollute or damage the air, soil, water, or animals. The sustainability of walnut production is influenced by factors such as its carbon footprint, water consumption, and impact on biodiversity and ecosystems [117]. The invasion of walnut trees into forest ecosystems can have multifaceted impacts, including unexpected linkages among invasive plants, native dispersers, land management, and topography, leading to cascading changes in ecosystems. Human activities, such as the intensive collection of walnuts for consumption or sale, can affect the dynamics of walnut fruit forests and their biodiversity. It takes around 0.76 kg CO_2_ eq to produce 1 kg of walnuts, indicating a relatively low carbon footprint compared with other edible nuts [119]. The water footprint of walnuts is moderate as walnuts require a significant amount of water for production compared to other nuts. Organic walnut production, when free of chemical factors such as fertilizers and plant protection products, is considered a sustainable investment. From an ecological perspective, walnut trees can improve soil structure and serve as alternatives to destructive land use [117]. The green walnut, as part of the walnut tree, has a low carbon footprint and water use relative to other plants because of the utilization of its various parts that might otherwise be considered waste and the intricate relationship between water and carbon, which influences tree growth and resource use.

Sustainable harvesting practices for green walnuts focus on minimizing their environmental impact and ensuring the long-term viability of walnut production. The following practices contribute to the sustainability of green walnut harvesting.

### 6.1. Selective Harvesting

Harvesting methods that avoid damage to the tree and promote natural reproduction include hand harvesting or the use of machinery that minimizes the impact on the surrounding environment.

### 6.2. Organic and Fair-Trade Certification

Choosing walnuts that are certified organic and fair trade can ensure that the products are produced with minimal environmental impact and under fair labor conditions [104].

### 6.3. Environmental Stewardship

Supporting research and innovation in water quality and conservation, soil health, energy use, and air quality can help minimize the environmental impact of walnut production [97].

### 6.4. Carbon Sequestration

Black walnut trees have potential for carbon sequestration, which can contribute to their sustainability. It also helps to increase the carbon content of soil and nourish soil nutrition [118]. It also aids in improving the soil [95,99].

### 6.5. Reduced Resource Consumption

Precision walnut farming aims to optimize the yield per acre through meticulous planning, cutting-edge technology, and sustainable practices, which can help reduce resource consumption and the environmental footprint of farming. By incorporating these practices, green walnut harvesting can be conducted in a sustainable manner, minimizing its environmental impact and ensuring the long-term viability of walnut production.

### 6.6. Use of Walnut Husk As Biomass Material

Research on the ecological uses of green walnut husk biomass has only begun, with an emphasis on the energy potential of this agricultural waste product and the recovery of valuable chemicals. Walnut green husks, usually discarded after walnut harvests, have been recognized to contain valuable components such as glucans and pectin’s and are being explored as a potential alternative fuel source because of their energy characteristics.

Nut shells, such as walnut husks, have energy qualities that make them suitable as alternative fuels, showing increased heating values following thermal processes, such as pyrolysis [120]. Differences in biomass generation capacity have been observed across various walnut cultivars, particularly in the distribution of green husk and in-shell biomass, potentially affecting their ecological applications [121].

### 6.7. Walnut Cultivation for Farmers and Producers

The use of green walnuts instead of ripe walnuts for human consumption offers various advantages to farms and farmers. Green manure from walnuts can greatly improve soil quality. Green manure has been proven to enhance soil water content, organic carbon, total nitrogen, and accessible phosphorus, which are crucial for the optimal growth of walnut trees and other crops [122]. Additionally, some green manures, such as hairy vetch, have been shown to enhance soil enzyme activity, reflecting a robust soil microbial population [122]. Implementing highly effective and integrated management technologies in walnut plantations, such as utilizing green walnuts, can reduce labor, enhance land productivity, and boost early economic gains. This method also helps reduce conflicts among agriculture, forestry, and animal husbandry, promoting the balanced growth of these industries [120]. Green walnut husks, often seen as agricultural by-products, have been recognized as a useful resource for extracting important chemicals, including glucans and pectin’s. These chemicals have diverse industrial uses and can be obtained from green husks, thus creating an extra source of income for growers [123].

Farms and producers are attracted to using green walnuts because of their benefits, such as enhancing soil health and fertility, aiding in poverty reduction and economic development, promoting efficient land management, and offering an opportunity for extra income through the extraction of valuable compounds from green walnut husks.

### 6.8. Role of Regulatory Approvals for Use of Green Walnuts

The search results did not provide information on the regulatory approval of green walnuts for human consumption, such as FDA approval or endorsements from international regulatory agencies. Conversations about walnuts, including green walnuts, typically center on their health advantages and culinary applications and the isolation of substances for scientific investigation (such as the antibacterial qualities of walnut green husks) [124,125]. The FDA’s produce safety rule set science-based minimum criteria for producing, harvesting, packing, and storing food for human consumption within the regulatory framework of food safety [126]. Although it does not explicitly mention green walnuts, this suggests that if green walnuts adhere to these broad safety criteria, they would be deemed safe for ingestion. To obtain a conclusive answer regarding green walnuts, it is necessary to directly contact regulatory agencies or review the relevant regulations pertaining to nuts and related products.

## 7. Industrial Applications

Green walnuts have various industrial applications including the production of condiments, pickles, sweets, preserves, and liqueurs. The industrial uses of walnut husks include the removal of hazardous materials and the extraction of biomass compounds. The by-products of walnut processing contain beneficial compounds that can be used in different fields [95]. Green walnuts, particularly their husks, have various industrial applications because of their chemical composition and functional properties [127]. It has been used in several applications for the preparation of polymer composites owing to its chemical composition, which includes ash, lignin, hemicellulose, and cellulose [108,109]. Natural dye source extracts from walnut husks can be used as natural dyes for dyeing materials such as polyamide fabrics [10] and for the removal of hazardous materials such as synthetic dyes and heavy metals from industrial effluents [115,116]. Green walnut farming and agroforestry can contribute to sustainable agriculture by providing diverse products, improving soil health, generating income, and providing environmental benefits. These practices can be adapted to various regions and agricultural systems, making them valuable components of sustainable agriculture [103]. Green walnut farming and agroforestry can play significant roles in sustainable agriculture by combining the cultivation of walnut trees with other agricultural practices, such as haymaking and beekeeping. Agroforestry systems can provide a wide range of products, including nuts, fruits, hay, and bees, which can contribute to the overall sustainability of farms. Walnut orchards can benefit from the use of cover crops such as annual reseeding grasses and legumes, which can be established in all walnut orchards. These cover crops can provide many benefits, including improved soil health, weed suppression, and support for an integrated pest management system [128]. Walnut trees can improve soil structure and fertility, making them an alternative to low-paying and environmentally destructive practices, such as cattle ranching [129]. Walnut growers can employ Integrated Pest Management strategies to reduce the use of chemical pesticides and herbicides and instead use natural fertilizers such as compost and cover crops [130]. Walnut cultivation can provide additional income for farmers as walnuts are a valuable crop with growing market demand. Agroforestry systems can help maintain good public health, improve quality of life in rural areas, and contribute to the conservation of natural resources [131].

Combining apple and walnut tree orchards with haymaking, beekeeping, and other agricultural practices. Organic walnut cultivation in intensive and super-intensive systems can be a sustainable investment in regions with suitable environmental conditions. Walnut production can be integrated into existing agricultural systems, such as the walnut fruit forests of southern Kyrgyzstan, where agroforestry and hay and walnut production complement each other.

## 8. Challenges and Future Prospects

The utilization of green walnuts poses multifaceted challenges across culinary, health, regulatory, and industrial domains. The inherent bitterness of green walnuts, which is attributed to compounds such as juglone and tannins, presents a taste hurdle that requires meticulous management. Moreover, the delicate nature of the soft shell and inner husk requires specialized processing techniques to extract the nut without compromising its integrity. However, the short shelf life of green walnuts coupled with seasonal availability limits their use and necessitates timely consumption or preservation methods. Health concerns arise from potential allergies to green walnuts and their constituents, warranting careful consideration. Regulatory challenges include addressing the impact of juglone on the environment and other crops, posing potential obstacles to its widespread adoption. Industrially, the limited use of green walnuts beyond niche culinary applications is due to processing costs and challenges in maintaining consistent quality. Sustainability concerns revolve around the ecological impact of harvesting and the need for responsible waste management. Despite these challenges, ongoing research and technological advancements offer promising avenues for overcoming these obstacles and tapping into the diverse potentials of green walnuts across various industries.

## 9. Conclusions

The green walnut (*Juglans regia* L.), a plant with significant ecological and economic value, has many applications and benefits. Its ecological importance lies in its capacity as a medicinal plant, its nutritional content, and its potential contribution to climate change-mitigation strategies. The green husk of the walnut fruit is particularly rich in phenolic compounds which exhibit antioxidant and antimicrobial properties, making it a promising candidate for treating diseases such as diabetes, rheumatic pain, cardiovascular diseases, and skin diseases. The use of green walnuts and their by-products extends to various industries, including the food and beverage, pharmaceutical, culinary, livestock feed, and textile industries. Walnut production has a relatively low carbon footprint. However, it is crucial to consider its water consumption and potential impacts on biodiversity and ecosystems. The adoption of organic cultivation practices can contribute to the sustainability of walnut production. Green walnuts possess high nutritional value and bioactive compounds, making them suitable for various industrial applications. Agroforestry models and practices can promote environment-friendly methods, reduce the use of chemical inputs, and enhance soil health and productivity, thereby contributing to sustainable agriculture. Additionally, the adaptability and relevance of green walnuts in environmental management make them a valuable resource for future research and conservation.

## Data Availability

All data were collected from published research papers.

## Abbreviations

FAO	Food and Agriculture Organization
WGH	Walnut green husk
WSP	Walnut septum
WMBPs	Walnut meal bioactive peptides
WM	Walnut meal
